# Chemical structure and pharmacokinetics of novel quinolone agents represented by avarofloxacin, delafloxacin, finafloxacin, zabofloxacin and nemonoxacin

**DOI:** 10.1186/s12941-016-0150-4

**Published:** 2016-05-23

**Authors:** Bela Kocsis, J. Domokos, D. Szabo

**Affiliations:** Institute of Medical Microbiology, Semmelweis University, Nagyvárad tér 4, Budapest, 1089 Hungary

**Keywords:** Chemical structure, Clinical trials, Pharmacokinetics, Quinolones, Safety, Tolerability, Toxicity

## Abstract

Quinolones are potent antimicrobial agents with a basic chemical structure of bicyclic ring. Fluorine atom at position C-6 and various substitutions on the basic quinolone structure yielded fluoroquinolones, namely norfloxacin, ciprofloxacin, levofloxacin, moxifloxacin and numerous other agents. The target molecules of quinolones and fluoroquinolones are bacterial gyrase and topoisomerase IV enzymes. Broad-spectrum and excellent tissue penetration make fluoroquinolones potent agents but their toxic side effects and increasing number of resistant pathogens set limits on their use. This review focuses on recent advances concerning quinolones and fluoroquinolones, we will be summarising chemical structure, mode of action, pharmacokinetic properties and toxicity. We will be describing fluoroquinolones introduced in clinical trials, namely avarofloxacin, delafloxacin, finafloxacin, zabofloxacin and non-fluorinated nemonoxacin. These agents have been proved to have enhanced antibacterial effect even against ciprofloxacin resistant pathogens, and found to be well tolerated in both oral and parenteral administrations. These features are going to make them potential antimicrobial agents in the future.

## Background

Quinolones are potent synthetic antimicrobials first developed in the 1960s. Since then several agents have been synthetised by modification of basic bicyclic chemical structure. Quinolones and fluoroquinolones are classified based on their chemical structure, antibacterial spectrum and pharmacokinetic features. Each agent inhibits bacterial DNA synthesis by forming a ternary complex with a DNA molecule and gyrase and topoisomerase IV enzymes, thus blocking bacterial DNA supercoiling [1–3].

The first quinolone agents were nalidixic acid, cinoxacin and oxolinic acid, each had basic bicyclic quinolone ring. These agents achieved 20–40 mg/L peak serum concentrations (C_max_) after a treatment with doses of 500–1000 mg. These agents and their metabolites were excreted by kidney and they reached 500–1000 mg/L peak urine concentrations 2–4 h following adminstration. The narrow-spectrum activity of these quinolones limited their use in clinical practice [4, 5].

Substituents on certain part of quinolone ring can increase potency of agents namely, in position C1 cyclopropyl or difluorophenyl, in position C6 a fluorine and in position C8 a halogen, metoxy or fused third ring. Quinolones harbouring a piperazin in position C7 are more effective on Gram-negatives and target topoizomerase IV. Agents targeting both gyrase and topoisomerase IV result have broad-spectrum effect [5].

The addition of fluorine and other substituents on the basic quinolone structure yielded fluoroquinolones, namely ofloxacin, ciprofloxacin, norfloxacin, pefloxacin, levofloxacin, moxifloxacin and several additional agents. These structural changes enhanced their tissue penetration as they achieved therapeutic concentrations in kidney, lung and intestine. Besides improved pharmacokinetic parameters, activity spectrum of these agents was also enhanced, as they showed bactericidal effect against numerous pathogens including Gram-positives, Gram-negatives, aerobes and anaerobes, moreover, antibacterial effect of fluoroquinolones is considered to be concentration-dependent [6, 7].

Ciprofloxacin is the most widely used fluoroquinolone agent with a potency against Gram-negatives. Levofloxacin (stereoisomer of ofloxacin) has bactericidal effect against Gram-negative and Gram-positive pathogens. Moxifloxacin is characterized by antibacterial effect mainly against Gram-positives including anaerobes, although they lack potency against Gram-negative anaerobes (e.g.: *Bacteroides* sp.) [8].

Gemifloxacin has also antibacterial activity against Gram-positive anaerobes. Garenoxacin, lacks fluorine in position 6, thus belonging to desfluoroquinolone group [2].

Despite the fact that numerous fluoroquinolone agents have been produced in the last decades, only a few of them are marketed, and some of them have been withdrawn or restricted because of their toxicity [7]. The most frequent reasons for withdrawal included tendinitis after treatment with pefloxacin; rashes appeared after sparfloxacin and clinafloxacin therapy; electrocardiogram disorders such as QTc prolongation occured during grepafloxacin administration; gatifloxacain and clinafloxacin therapy led to dysglycemia; hemolysis occured during temafloxacin administration; hepatotoxicity was found in trovafloxacin treatment [2, 7, 9]. The pharmacokinetic properties of quinolones are listed in Table 1.

**Table 1:** Pharmacokinetic features of quinolones

Quinolone agents	Protein binding (%)	Urinary fraction (%)	Bioavailability (%)	C_max_ (mg/L)	t_1/2_ (h)	Ref.
Ciprofloxacin	20–40	40–50	70	4.3	4	[6]
Levofloxacin	24–38	87	99	6.2	6–7	[6]
Sparfloxacin	45	10	92	1.1	20	[6]
Trovafloxacin	76	6	88	2.1	9.6	[6]
Moxifloxacin	50	20	90	4.5	12	[6]
Gatifloxacin	20	72	96	3.8	7.8	[6]
Avarofloxacin	65	12	65	2	14	[12]
Delafloxacin	16	n.a.	n.a.	10	12	[39]
Finafloxacin	n.a.	33	n.a.	11	10	[19]
Zabofloxacin	n.a.	n.a.	n.a.	2	8	[27]
Nemonoxacin	16	n.a.	n.a.	5	15	[40]

*Ref* reference number

*Urinary fraction* urinary fraction excreted unbound

*C*_*max*_ peak serum concentration

*t*_*1/2*_ half-life time

*n.a.* not available

In the past years, identification of new molecules were in focus to obtain antibacterial agents with potency against pathogens that already developed resistance to fluoroquinolones. Structure–activity relationship studies played key role to detect substituents that had high affinity for binding to both DNA gyrase and topoizomerase IV enzymes. Among developed agents five are undergoing clinical testing and all showed enhanced antibacterial activity including strains exhibiting resistance to present-day fluoroquinolones. These agents are avarofloxacin (JNJ-Q2), delafloxacin (WQ-3034), finafloxacin (BAY35-3377), zabofloxacin (DW224a) and non-fluorinated nemonoxacin (TG-873870).

**Fig. 1:**
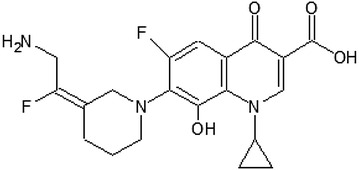
Avarofloxacin

*Avarofloxacin* (JNJ-Q2) (Fig. 1) is an aminoethylidenylpiperidine fluoroquinolone with a zwitterion structure that demonstrates antibacterial effect against numerous Gram-positive bacteria with a 0.12 mg/L MIC_90_ value, therefore it is found to be more potent than previously used fluoroquinolones. Tested pathogen bacteria included strains of *Streptococcus pneumoniae,* methicillin-resistant *Staphylococcus aureus* (MRSA), *Enterococcus* sp., *Escherichia coli, Klebsiella* spp., *Haemophilus influenzae* and *Pseudomonas aeruginosa* [10] (Table 2). Besides, avarofloxacin showed a potent antibacterial effect against *Neisseria gonorrhoeae* with a 0.25 mg/L MIC_90_ value, compared to 16 mg/L of ciprofloxacin [11].

**Table 2:** Quinolone MIC values of medically relevant pathogens

Organism	Antibacterial agents	MIC range	MIC_90_	Ref.
*S. pneumoniae*	Avarofloxacin	0.06–0.5	0.25	[10]
	ciprofloxacin	8–64	64	[10]
	Delafloxacin	n.a.	n.a.	n.a.
	Finafloxacin	0.5–4	2	[41]
	Zabofloxacin	0.015–0.06	0.03	[24]
	Nemonoxacin	0.03–1	0.06	[34]
*S. aureus,* MRSA FQ-resistant	Avarofloxacin	0.015–2	0.25	[10]
	Ciprofloxacin	4 ≥ 256	64	[10]
	Delafloxacin	≤0.004–0.12	0.06	[15]
	Finafloxacin	0.25–32	4	[41]
	Zabofloxacin	0.016–64	32	[26]
	Nemonoxacin	0.5–1	1	[34]
*S. aureus*, MRSA FQ-susceptible	Avarofloxacin	0.008–0.015	0.008	[42]
	Ciprofloxacin	0.25–1	0.5	[42]
	Delafloxacin	0.008–1	0.5	[13]
	Finafloxacin	0.06–0.125	0.125	[41]
	Zabofloxacin	0.016–1	0.125	[26]
	Nemonoxacin	≤0.008–0.12	0.06	[31]
*E. faecalis*	Avarofloxacin	0.03–1	0.5	[10]
	Ciprofloxacin	0.5–>16	>16	[10]
	Delafloxacin	n.a.	n.a.	n.a.
	Finafloxacin	1–2	n.a.	[22]
	Zabofloxacin	0.008 ≥ 4	2	[24]
	Nemonoxacin	0.12–8	4	[34]
*E. faecium*	Avarofloxacin	0.25–4	4	[10]
	Ciprofloxacin	1 ≥ 16	>16	[10]
	Delafloxacin	n.a.	n.a.	n.a.
	Finafloxacin	n.a.	n.a.	n.a.
	Zabofloxacin	2–32	16	[24]
	Nemonoxacin	0.06–16	16	[34]
*E. coli*	Avarofloxacin	1–16	16	[10]
	Ciprofloxacin	16 ≥ 256	256	[10]
	Delafloxacin	2–128	n.a.	[43]
	Finafloxacin	16 ≥ 256	256	[41]
	Zabofloxacin	0.015–64	1	[24]
	Nemonoxacin	0.5–32	32	[31]
*K. pneumoniae*	Avarofloxacin	≤0.015–1	0.25	[10]
	Ciprofloxacin	≤0.004–1	0.25	[10]
	Delafloxacin	n.a.	n.a.	n.a.
	Finafloxacin	0.015–0.6	n.a.	[22]
	Zabofloxacin	0.06–8	1	[24]
	Nemonoxacin	0.5–32	2	[31]
*P. aeruginosa*	Avarofloxacin	0.5–4	2	[10]
	Ciprofloxacin	0.12–1	0.5	[10]
	Delafloxacin	0.016–1	n.a.	[43]
	Finafloxacin	0.25–8	2	[41]
	Zabofloxacin	<0.008 ≥ 64	>64	[24]
	Nemonoxacin	0.12–32	32	[31]
*H. influenzae*	Avarofloxacin	0.008–0.015	0.015	[10]
	Ciprofloxacin	0.008–0.015	0.015	[10]
	Delafloxacin	n.a.	n.a.	n.a.
	Finafloxacin	n.a.	n.a.	n.a.
	Zabofloxacin	<0.008–0.008	0.008	[24]
	Nemonoxacin	≤0.008–0.06	n.a.	[38]

All values are in mg/L

*Ref* reference number

### Pharmacokinetics

Avarofloxacin is applicable both in *per os* and in *parenteral* administration. In the case of parenteral dosing of 90 min avarofloxacin serum concentration declines biexponentially with a short distribution phase and an extended terminal phase. During oral dosing the concentration decreased monoexponentially. Mean half-life time of agent was found similar for 15 and 30 mg doses 13.4 and 12.9 h, respectively. In the case of 75 and 150 mg doses showed 15.1 and 16.7 h. A single 250 mg oral avarofloxacin dose reached its C_max_ in 2.18 mg/L 2 h after administration. The bioavailability of avarofloxacin is 65–66 % in parenteral—oral administration [12].

### Toxicity

Avarofloxacin was well tolerated during single intravenous (iv) administration up to the maximum dose of 150 mg. Frequent, mild adverse events were observed including headache and contact dermatitis. All adverse events were grade I including a transient diarrhea and lipase elevation after administration of 75 mg, while phlebitis appeared after a 15 mg iv dose.

Multiple iv doses were also well tolerated up to 150 mg twice daily adminstration, as nausea, vomiting, diarrhea, headache and chills appeared [12].

**Fig. 2:**
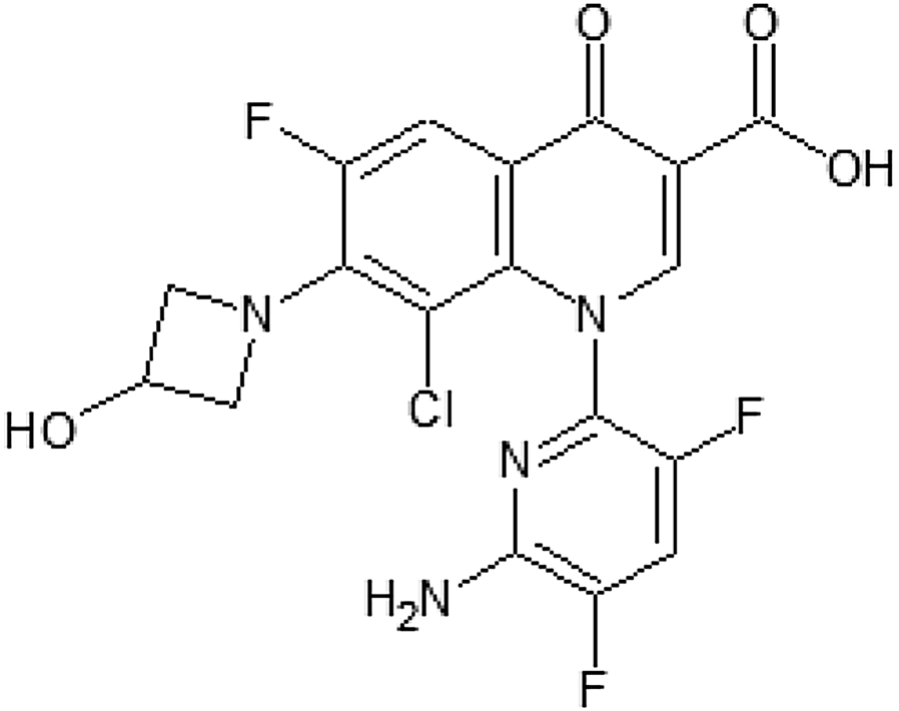
Delafloxacin

*Delafloxacin* (WQ-3034) (Fig. 2) has a chemical structure of 1-(6-amino-3,5-difluoro-2-pyridinyl)-8-chloro-6-fluoro-7-(3-hydroxy-1-azetidinyl)-4-oxo-1,4-dihydro-3-quinolinecarboxylate, which differs in three features from classical fluoroquinolones: in position C7 it lacks a strongly basic group this confers weak acidity; in position C8 a chlorine exhibits a strong electron-withdraw on aromatic ring; in position N1 a heteroaromatic substitution leads to a larger molecular surface compared to current fluoroquinolones [13]. At neutral pH, delafloxacin exists in a deprotonated form [14]. Delafloxacin targets both DNA gyrase and topoisomerase IV enzymes making it a potent agent. The anionic structure of delafloxacin appears to enhance its potency in an acidic environment, therefore its antibacterial activity is increased in environments with reduced pH (e.g.: phagolysosome, inflammatory cells) or in skin and soft tissue infections of *S. aureus.* This feature makes delafloxacin special among fluoroquinolones as ciprofloxacin and moxifloxacin have less activity in acidic sites [14, 15]. Besides its direct antibacterial effect, the inhibition of *S. aures* biofilm production was also detected [16].

### Pharmacokinetics

Efficacy of delafloxacin was analyzed in a phase 2, multicenter, randomized, double-blind study. Delafloxacin’s antibacterial effect was compared to tigecycline in skin and soft tissue infections of 150 patients. Two different iv doses of delafloxacin of 300 and 450 mg were administered every 12 h and compared to tigecycline given iv in doses of 100 mg plus iv 50 mg every 12 h. The study was performed for 5–14 days based on clinical outcome. No significant differences were found between the three treatment options as each was effective in both *S. aureus* and MRSA skin and soft tissue infections. The ciprofloxacin MIC values of the pathogens ranged between 0.12 and 32 mg/L, while for delafloxacin it varied between 0.004 and 0.12 mg/L [15].

A phase 1 single-dose study analyzed efficacy of delafloxacin under different feeding conditions. Altogether 30 healthy individuals were enrolled where each sequence comprised 3 treatments of a single *per os* dose of 900 mg delafloxacin under fasting conditions for at least 10 h (group A), under fed conditions of standardized FDA high fat breakfast 30 min before dosing (group B) and in fasting followed by a high fat meal 2 h after dosing (group C). The pharmacokinetic parameters of delafloxacin was analyzed in each group; C_max_ was 11.5, 9.14 and 11.8 mg/L in the corresponding group. The time to reach C_max_ was found 1.25, 2.5 and 1.5 h while half-life time of delafloxacin was 14.1, 12.9 and 12 h, respectively [17].

### Toxicity

Delafloxacin was well-tolerated during the multicenter study, although adverse events of nausea, diarrhea, headache, insomnia and fatigue appeared in iv administered 300 mg and 450 mg delafloxacin groups [15].

In phase 1 single dose trial, delafloxacin was also well tolerated but following adverse events appeared in A, B and C groups: diarrhea, nausea, presyncope, headache, vaginal infections or pharyngitis [17].

**Fig. 3:**
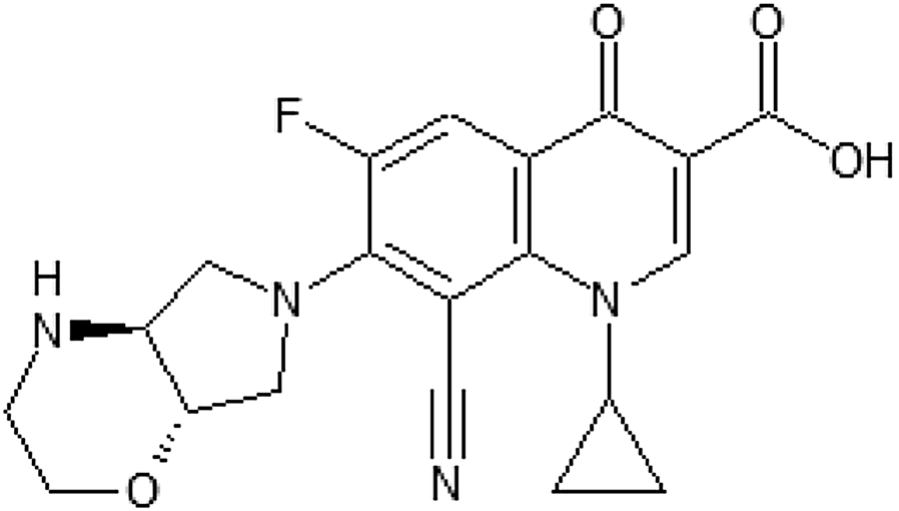
Finafloxacin

*Finafloxacin* (BAY35-3377) (Fig. 3) is a fluorinated quinolone derivative with 8-cyano-substituent and 7-pyrrolo-oxazinyl moiety. It has a zwitterion chemical structure with an isoelectric pH of 6.7 and two dissociation constants at a pK_a1_ of 5.6 (carboxylate function) and a pK_a2_ of 7.8 (nitrogen at C7 substituent), in contrast to ciprofloxacin with an isoelectric pH of 7.4 and two dissociation constants at pK_a1_ of 6.1 and pK_a2_ of 8.7 [18]. Finafloxacin has enhanced antibacterial activity under acidic conditions, this is unique among fluoroquinolones and advantageous in specific infection sites namely, skin and soft tissue, vagina and urinary tract. The maximum bactericidal activity was observed at pH 5–6. However, at neutral pH the antibacterial activity was similar to the previously used fluoroquinolones [19]. These properties of finafloxacin could offer advantage in treatment of infections in acidic anatomical sites or in inflammatory processes in acidic environment, such as respiratory and urinary tracts, skin and intraabdominal sites. Antibacterial activity in acidic pH conditions might be benefitial in *Helicobacter pylori* eradication too [20]. Furthermore, antibacterial effect of finafloxacin against phagocytized *Legionella pneumophila* and *Listeria monocytogenes* was investigated. Finafloxacin was found to be more potent against *L. pneumophila* than ciprofloxacin, although against *L. monocytogenes* it was less effective [21].

### Pharmacokinetics

Plasma pharmacokinetics of finafloxacin was analyzed after orally administered single doses of 25, 50, 100, 200, 400 and 800 mg. The C_max_ values were: 0.24, 0.44 ± 0.16, 1.32 ± 0.62, 1.90 ± 0.73, 5.06 ± 2.09 and 11.1 ± 2.96 mg/L, respectively. The half life-time of finafloxacin in single dose administration were as follows: 1.28, 3.8 ± 2.7, 7.2 ± 3.2, 4.6 ± 1.9, 10.0 ± 4.4 and 10.5 ± 2.2 h, respectively. The time to reach the C_max_ varied between 0.5 and 1 h.

In the case of multiple doses of 150, 300, 600 and 800 mg were administered orally once daily for seven consecutive days. The C_max_ after the seventh day showed the following concentrations: 1.50 ± 0.52, 4.15 ± 2.11, 6.76 ± 2.2 and 8.95 ± 3.11 mg/L. The corresponding half-life time was 5.3 ± 0.6, 6.5 ± 2.5, 8.8 ± 3.1 and 14 ± 5.5 h, respectively. While time to reach C_max_ was between 0.5 and 1.5 h [19].

Accumulation of finafloxacin was not relevant following daily once application of doses up to 800 mg over 7 days. Peak serum concentrations may slightly increase proportionally compared to dose [19].

The urine recovery data show that around 30 % of oral dose can be detected. Moreover, the urine finafloxacin concentration exceeds MIC values determined in Mueller–Hinton broth for many pathogens of urinary tract infections e.g.,: *E. coli* and *P. aeruginosa*. This feature makes it advantageous in treatment of several urinary tract infections [21, 22].

The mean concentration of finafloxacin in urine was 68 mg/L in the first 4 h and 4 mg/L at the time of 12–24 h sampling following 200 mg dose. In the case of the 800 mg dose the mean finafloxacin concentration was 112 mg/L in the first 4 h (peak of 150 mg/L was reached in the 4–8 h interval) and 18 mg/L in the 12–24 h sampling period [23].

### Toxicity

Finafloxacin is found to be well-tolerated agent, although minor adverse events were detected such as central nervous system events including headaches. Gastrointestinal and respiratory disorders appeared, namely diarrhea, loose stool, nausea, flatulence, rhinitis and nasopharyngitis. Frequency of adverse events did not vary between the different administered doses and their corresponding placebo groups, except for gastrointestinal events, as they appeared during actively treated subjects [19].

**Fig. 4:**
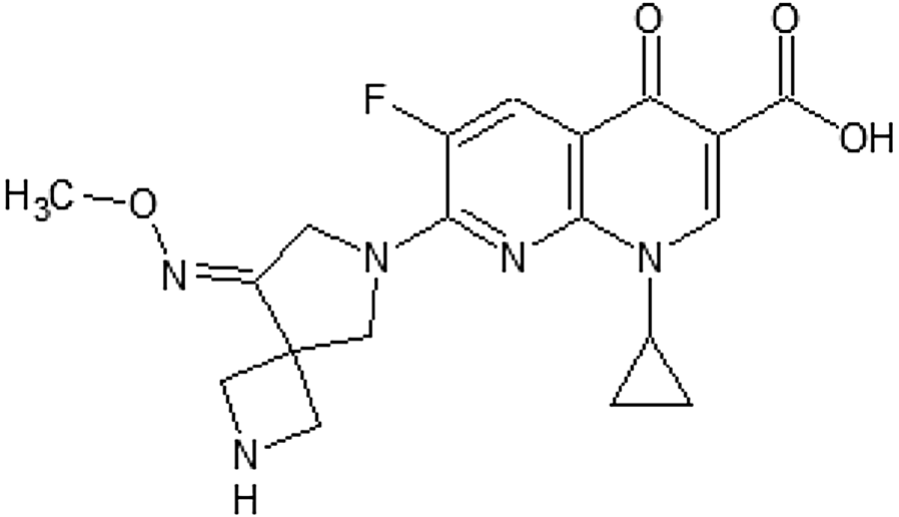
Zabofloxacin

*Zabofloxacin* (DW-224a) (Fig. 4) is a broad-spectrum fluoroquinolone as it achieves bactericidal effect against various Gram-positive and Gram-negative pathogens, even against fluoroquinolone resistant ones [24–26]. Two formulations of zabofloxacin are available, namely zabofloxacin hydrochloride (DW-224a) and aspartate (DW-224aa) [27].

### Pharmacokinetics

Zabofloxacin hydrochloride and aspartate were analyzed in a random, open-label single-dose study with enrollment of twenty-nine healthy males. The pharmacokinetic parameters were set after oral adminstration of 366.7 mg zabofloxacin hydrochloride and 366.5 mg zabofloxacin aspartate. C_max_ values were 1.9 ± 0.5 and 2 ± 0.3 mg/L and these concentrations were reached in plasma between 0.5 and 4 h and 0.8–3 h, respectively. The half-life time of zabofloxacin was 8 ± 1 h for both formulations [27].

Pharmacokinetic parameters of zabofloxacin hydrochloride were analyzed in beagle dogs. Orally administered zabofloxacin hydrochloride was given in 10, 30 and 90 mg/kg/day for a total of 4 weeks. The absorption of the agent was fast, as 30 min after administration plasma zabofloxacin concentration was detected. C_max_ was 10 mg/L and time to reach it was 1 h [28].

Zabofloxacin pharmacokinetic parameters were also investigated in a rat model. 20 mg/kg single dose zabofloxacin hydrochloride was orally administered to the animals. C_max_ was 1.8 ± 0.8 mg/L and it was reached within 33.8 ± 18.9 min. The half-life time of zabofloxacin was 107 ± 13.3 min [29, 30].

### Toxicity

Single oral dose of both formulations of zabofloxacin were found to be well-tolerated among healthy male volunteers. The most frequent adverse events were nausea (7 % of the subjects), hypotension (3 %), somnolence (3 %), increase of blood phosphokinase (3 %). By contrast, prolongation of QT interval a typical adverse event of fluoroquinolones was not detected [27].

Subacute toxicity was analyzed in beagle dogs. All tested animals presented vomiting and salivation at 30 and 90 mg/kg/day doses, although only one subject showed these adverse events at 10 mg/kg/day dose. Anorexia, decreased food intake and body weight gain was detected in 90 mg/kg/day group during the 20th and the 28th days. Significant serum total cholesterol increase was detected in the 30 and 90 mg/kg/day group in the fourth week of the study. Electrocardiogram showed a trend toward increased QT intervals at 90 mg/kg/day groups. Atrophy of testicles was observed and consequently, oligo and aspermia were detected. Thymus, spleen and adrenal gland atrophy was found in 30 and 90 mg/kg/day doses [28].

**Fig. 5:**
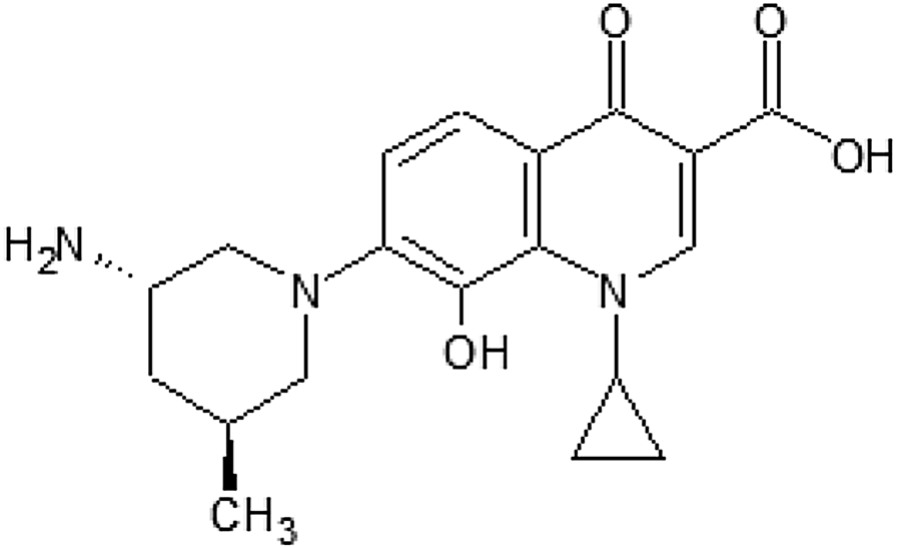
Nemonoxacin

*Nemonoxacin* (TG-873870) (Fig. 5) is a C-8-metoxy non-fluorinated broad-spectrum quinolone, generally more active than classic fluoroquinolones. The C-8-methoxy substituent on the quinolone ring increases antibacterial effectiveness against Gram-positives and reduces selection of resistant mutants. The lack of fluorine may decrease frequency of toxic adverse effects [31–33]. In vitro testing found that antibacterial activity of nemonoxacin is better against methicillin susceptible and resistant *S. aureus* [34].

### Pharmacokinetics

Pharmacokinetic parameters of nemonoxacin were investigated in a multiple-parameter study in healthy subjects. 500 or 750 mg of nemonoxacin was administered parenterally during 1.5 and 2.25 h and rate of 5.56 mg/min, once daily for 10 days continuously. In a randomized double-blind placebo controlled study 500, 650 or 750 mg of nemonoxacin or placebo was administered to healthy individuals parenterally during of 2 or 2.6 or 3 h at a rate of 4.17 mg/min, once daily for 10 days continuously [35]. Blood and urine nemonoxacin concentrations were analyzed by validated liquid chromatography–tandem mass-spectrometry (LC–MS/MS).

In the first stage, the maximal nemonoxacin concentrations were 9.6 ± 1.84 and 11 ± 2.2 mg/L during the administration of 500 and 750 mg whereas areas under concentration–time curve between 0 to 24 h (AUC_0-24_) were 44.03 ± 8.62 and 65.82 ± 10.78 µg h/ml.

Nemonoxacin drug accumulation was not relevant during a 10 days’ administration of both 500 and 750 mg [35].

Two-compartment model was used to analyze nemonoxacin pharmacokinetic profiles in healthy volunteers. Nemonoxacin distribution volumes in central compartment were in range of 64.5–83.2 L, whereas in peripheral one they ranged between 24.7 and 40.9 L.

The distribution half-life time of nemonoxacin showed dose-dependency. In case of parenteral administration of 500 mg (at 5.56 mg/min rate) 2.42 h while in the 750 mg dose it increased to 3.37 h. Increase in the corresponding elimination half-life time was from 10.8 to 12.7 h. By contrast, a slight decrease in the intercompartment clearance was seen when nemonoxacin concentration increased as nemonoxacin clearance rates ranged between 12.0 and 14.6 L/h [35].

Adminstration of nemonoxacin was analyzed for both oral and parenteral 500 and 750 mg dosing. Interestingly, the iv nemonoxacin C_max_ (at infusion rate of 4.17 mg/min) was similar to the orally administered 500 mg nemonoxacin: 7.13 and 7.02 mg/L. In case of 750 mg administration C_max_ values for iv and oral dosing were: 9.96 and 9.13 mg/L [36].

### Toxicity

Safety and toxicity of nemonoxacin were tested in healthy volunteers. The highest tolerable dose during iv nemonoxacin administration was 1250 mg and the most suitable infusion rate was 5.56 mg/min. Transient and mild adverse events appeared, namely injection site reaction, erythematous rashes with or without pruritus and abnormal electrocardiogram T-wave. All the above mentioned adverse events vanished during application or within 2 h after application, except in two individuals, one who received a dose of 750 mg at 4.17 mg/min infusion rate and another person also from the 750 mg dose-group, at 8.33 mg/min infusion rate [37].

In a study conducted with orally administered 500 and 750 mg nemonoxacin, the 44.9 and 55.8 % of subjects produced treatment emergent adverse events. A control group was also included in this study, where 500 mg of levofloxacin was administered and 48.9 % of subjects showed adverse events. Diarrhea, dizziness, headaches appeared in each group [38].

## Conclusions

Several attempts were followed to recognize antibacterial agents with new chemical structures, although numerous novel agents are derived from current antibiotic classes. The newly discovered agents should demonstrate more potent antimicrobial activity, efficacy against resistant pathogens, yield improved safety profiles and show enhanced pharmacokinetics. Based on pharmacological experience it is possible to design new molecules with improved pharmacokinetic and pharmacodynamic features in old antibiotic classes. The sophisticated crystallographic methods and structure–activity relationship studies play crucial role in drug discovery to detect multiple targets of a known antibiotic class.

The novel quinolone agents detailed in this review offer an improved antibacterial effect compared to earlier classes of fluoroquinolones. These antibiotics proved to have enhanced bactericidal activity even against various ciprofloxacin pathogens this confirms their role in treatment of numerous infections. The improved tolerability, safety profile and decreased toxicity enable them to be used in clinical practice.

The doses of all antibiotics should be well established on pharmacokinetic and pharmacodynamic properties, thus reducing selection of resistant mutants. Still, more information are required regarding efficacy against multidrug-resistant pathogens. These resistant strains are main targets for new drugs. Resistance determinants are well understood, so new molecules can be designed on a molecular level to avoid the most common resistance mechanisms. Furthermore, it is necessary to explore targets to serve as basis for new antibacterial agents in the future.
